# Evaluation of blood flow on the remnant distal bowel during left-sided colectomy

**DOI:** 10.1186/s12957-018-1487-2

**Published:** 2018-09-13

**Authors:** Takayuki Ogino, Masaki Okuyama, Tomoki Hata, Junji Kawada, Miho Okano, Yongkook Kim, Toshimasa Tsujinaka

**Affiliations:** Department of Surgery, Kaizuka City Hospital, Hori 3-10-20, Kaizuka-shi, Osaka 597-0015 Japan

**Keywords:** Anastomotic leakage, Left-sided colectomy, Indocyanine green

## Abstract

Adequate blood flow in anastomosis is of paramount importance to prevent anastomotic leakage. However, it is sometimes difficult to predict the viability of the intestine during surgery. During left-sided colectomy, blood flow on the remnant distal bowel is supplied only from the middle and inferior rectal arteries. The blood backflow after the root ligation of the inferior mesenteric artery is often said to be kept up to promontorium levels; however, this premise is actually based on experience, without reliable evidence. Here, we introduce the intraoperative evaluation of blood flow on the remnant distal bowel during left-sided colectomy using an indocyanine green fluorescence technique.

## Introduction

Anastomotic leakage (AL) is one of the most dreadful complications occurring after surgery for colorectal cancer. The reported incidence rate of AL after colorectal surgery ranges from 3 to 19%. AL can cause increased morbidity, mortality, length of hospital stay, cost, and risk of cancer recurrence [1, 2]. Adequate blood flow in anastomosis is of paramount importance for the prevention of AL; however, it is sometimes difficult to predict the viability of the ischemic bowel during surgery. The intraoperative evaluation of the intestinal blood flow is mostly performed by gross findings such as color, peristalsis, pulsation, and/or bleeding. It is largely dependent on the surgeon’s skill and experience. Therefore, objective and accurate measurements of intestinal blood flow could reduce AL.

During left-sided colectomy with root ligation of the inferior mesenteric artery (IMA), the blood flow on the remnant distal bowel is supplied from the middle and inferior rectal arteries. The blood backflow is often said to be kept up to promontorium levels after the root ligation of the IMA; however, this premise is based on experience without any evidence. In clinics, excessive sacrifice of the remnant distal bowel due to lack of objective indicators of blood flow is a possibility.

Indocyanine green (ICG) has been used for several decades to evaluate cardiac output, liver function, liver blood flow, and ophthalmic blood flow [3]. In recent years, an ICG fluorescence technique with near-infrared light has become a promising technique for evaluating intestinal blood flow intraoperatively [4].

While several reports showed the evaluation of blood flow on the remnant proximal bowel during left-sided colectomy, only a few studies evaluated the remnant distal bowel [3, 4]. We introduce an evaluation of blood flow on the remnant distal bowel during left-sided colectomy using an ICG fluorescence technique.

## Technique presentation

**Fig. 1:**
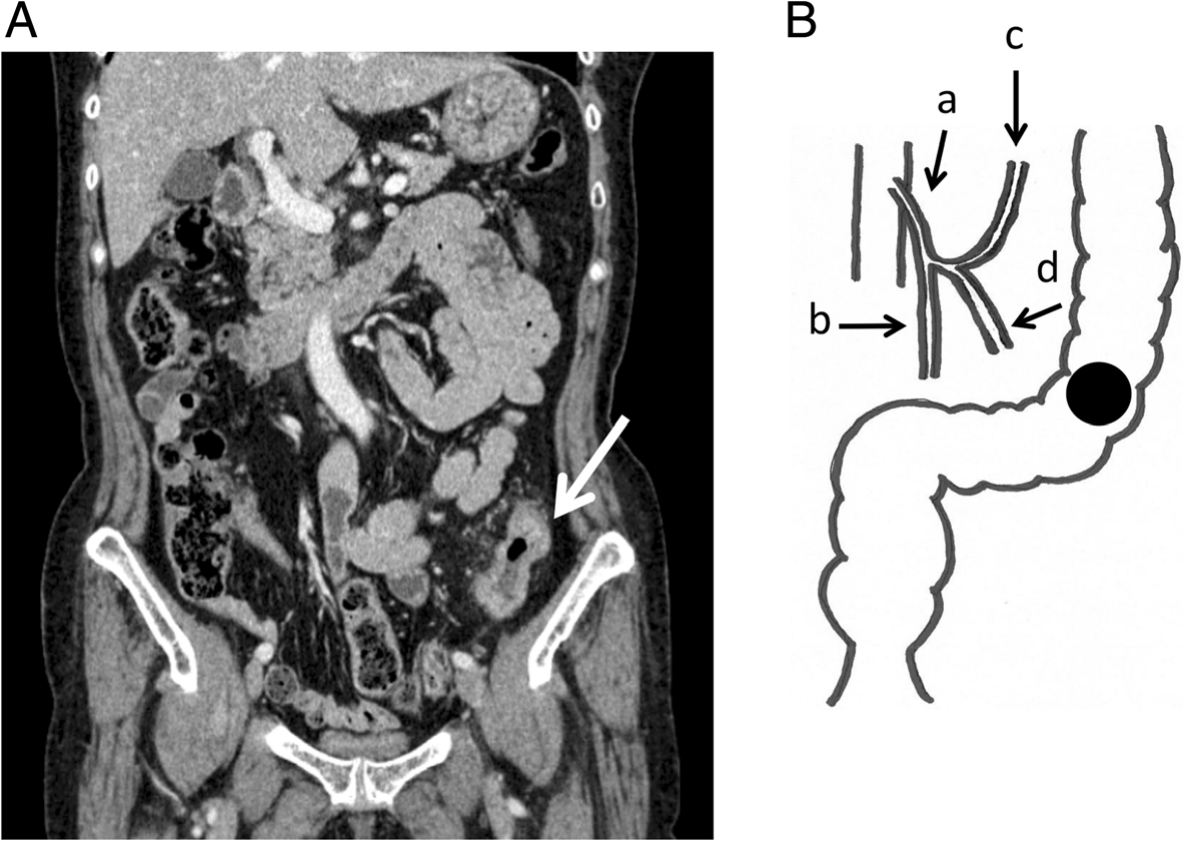
**A** The abdominal CT scan showed T3 colon cancer near the SD junction. The arrow indicates the tumor location. **B** Anatomy of this patient. a: inferior mesenteric artery, b: superior rectal artery, c: left colic artery, d: sigmoid artery

A 73-year-old female came to our hospital due to constipation. She had no medical history other than diabetes mellitus. A colonoscopy revealed sigmoid colon cancer. An abdominal computed tomography (CT) scan showed T3 colon cancer near the SD junction (Fig. 1). A laparoscopic sigmoidectomy was performed.

**Fig. 2:**
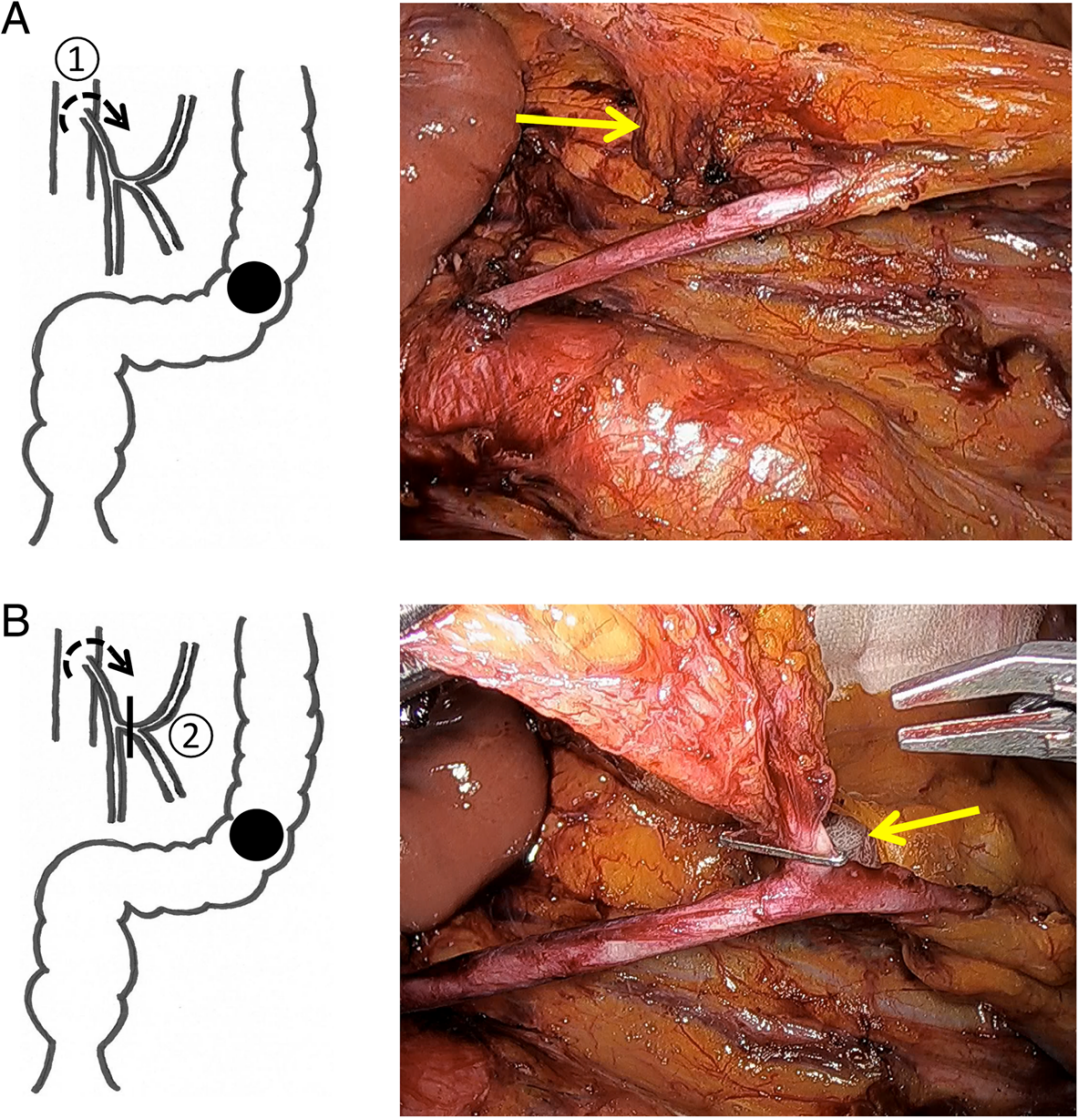
**a** Lymph node dissection around the root of the IMA (①, arrow) after exposing the IMA from the root to the distal side. **b** The common trunk of the left colic artery and sigmoid artery were ligated, with preservation of the superior rectal artery (②, arrow)

**Fig. 3:**
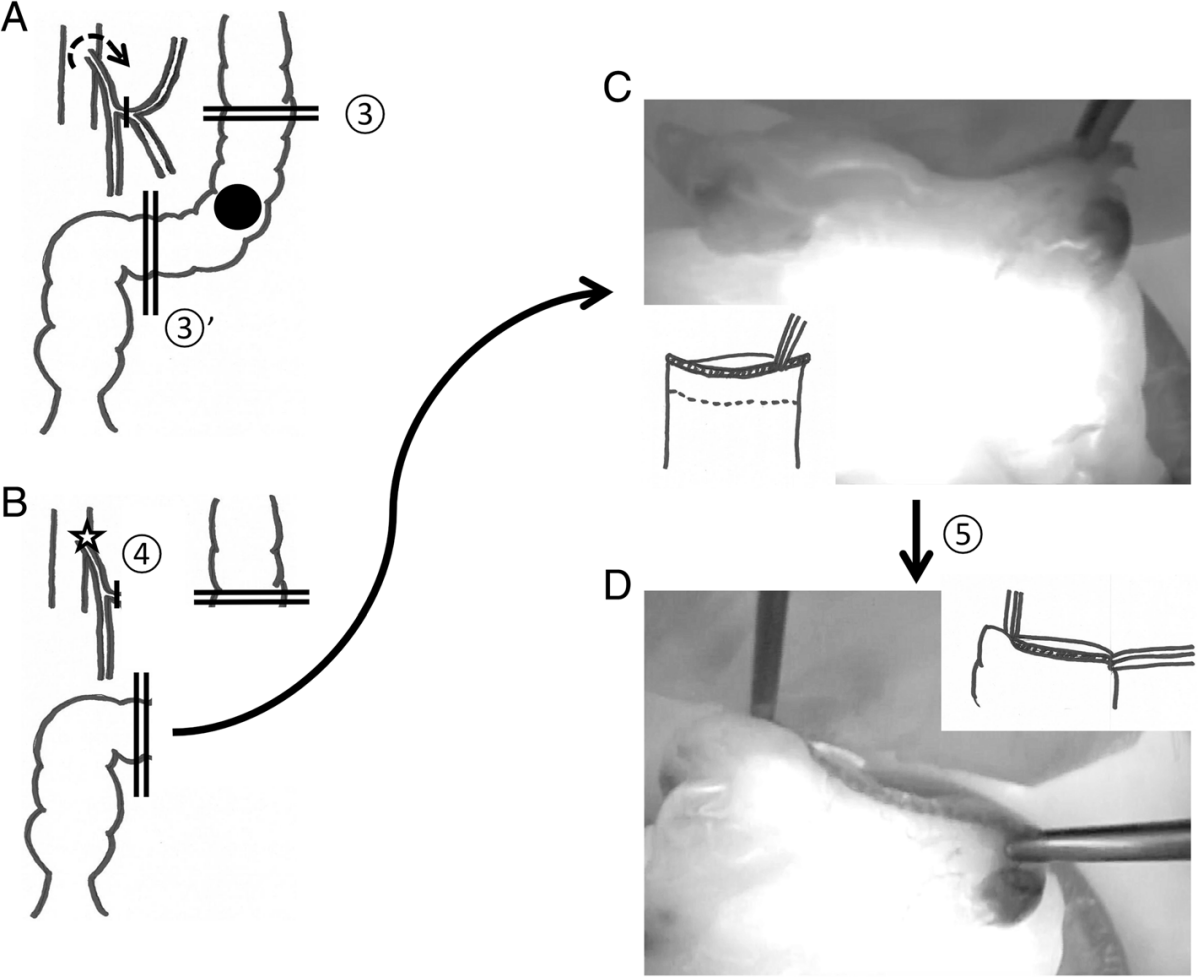
**a** The resected line of the proximal (③) and distal (③′) bowel was determined, with a 10-cm margin from the tumor. **b** Blood flow evaluation after the clamp test of the IMA (④, star mark) based on the presumption that the root ligation of the IMA was performed. **c** Blood flow around the remnant distal bowel stump was quite poor after clamping the IMA. **d** After the IMA clamp was released (⑤), the blood flow around the remnant distal bowel stump fully recovered

The intraperitoneal observation confirmed no liver metastasis and no peritoneal dissemination. The small intestine was moved to the upper right side to keep the surgical field open. The mobilization of the left side of the colon up to the splenic flexure and the mobilization of the rectum were performed using a medial approach. Lymph node dissection around the root of the IMA was performed, exposing the IMA from the origin to the distal side (Fig. 2a). The common trunk of the left colic artery (LCA) and sigmoid artery was ligated and lymph nodes were dissected around the root of the IMA, with preservation of the superior rectal artery (Fig. 2b). The proximal and distal bowel was resected, with a 10-cm margin from the tumor (Fig. 3a). The distal side of the resected bowel was located 3 cm proximal to the promontorium. Subsequently, we performed an intraoperative blood flow evaluation; ICG 5 mg/2 ml was administered intravenously, and the evaluation of the remnant bowel stump was performed before anastomosis using the NIR light camera system (Photodynamic Eye System, Hamamatsu Photonics, Japan). The blood flow on the remnant proximal bowel stump was fine. Next, we tried the blood flow evaluation following a clamp test of the IMA based on the presumption that the root ligation of the IMA was performed (Fig. 3b). The blood flow around the remnant distal bowel stump was quite poor during the IMA clamp (Fig. 3c). After the IMA clamp was released, the blood flow was fully recovered (Fig. 3d).

The patient was discharged without complications on postoperative day 10. Our case showed a morbid risk for AL if the root ligation of the IMA had been performed without the intraoperative blood flow evaluation on the remnant distal bowel.

## Discussion

The rate of lymph node metastasis around the root of the IMA is 3.6% in patients with T3/T4 sigmoid colon cancer [5]. In Japan, the surgical procedure, with the dissection of lymph nodes along with IMA and the ligation and division on the root of the LCA, is commonly performed. This procedure is based on the concept of the significance of the lymph node dissection around the root of the IMA by means of the root ligation of the IMA, as well as the significance of preserving anastomotic perfusion by preserving the superior rectal artery. In our case, the operation was also performed according to this concept.

Regarding the blood flow in the left side of the colon or rectum, several studies have reported mainly on Sudeck’s critical point. These reports anatomically clarified the continuity of blood flow in the remnant proximal bowel after left-sided colectomy with anatomical examinations using radiography or autopsy [6, 7]. The examination of the blood flow in the remnant distal bowel after left-sided colectomy was also conducted in a few studies. These publications reported the variation in the middle rectal artery and anatomically examined the perfusion area of the superior, middle, and inferior rectal arteries [8, 9]. However, no functional evaluation has been performed so far.

Our case revealed the possibility of preserving more of the remnant distal bowel using the ICG fluorescence technique. This technique also can be applied in cases where there is damage to marginal vessels during surgery or in cases with severe arterial sclerosis.

During left-sided colectomy, the distal side of the bowel was often excessively resected forcing the anastomosis to be performed at points lower than the promontorium. However, the lower anastomosis makes the procedure difficult to perform and may cause anastomotic failure. Preserving a longer remnant distal bowel is desirable to minimize anastomotic tension and promote postoperative bowel function if enough blood supply and the margin from the tumor can be confirmed. Larger studies of the ICG fluorescence technique are needed to confirm and verify the effects on AL.
